# Exosomes secreted by FNDC5-BMMSCs protect myocardial infarction by anti-inflammation and macrophage polarization via NF-κB signaling pathway and Nrf2/HO-1 axis

**DOI:** 10.1186/s13287-021-02591-4

**Published:** 2021-09-28

**Authors:** Hongjuan Ning, Haixu Chen, Jingyu Deng, Chun Xiao, Moyan Xu, Lina Shan, Chao Yang, Zheng Zhang

**Affiliations:** 1 The First Affiliated Hospital of Jinzhou Medical University, Jinzhou, 121001 China; 2 Institute of Geriatrics, Health Care Office, National Clinical Research Center of Geriatrics Disease, Chinese PLA General Hospital, Beijing, 100853 China; 3 Air Force Military Medical University, Xi’an, 710032 Shaanxi China; 4 PLA Rocket Force Characteristic Medical Center, Beijing, 100088 China

**Keywords:** Exosomes, Bone marrow mesenchymal stem cells (BM-MSCs), Myocardial infarction (MI), Fibronectin type III domain-containing protein 5 (FNDC5), Inflammation, Macrophage polarization

## Abstract

**Background:**

Exosomes are considered a substitute for stem cell-based therapy for myocardial infarction (MI). FNDC5, a transmembrane protein located in the cytoplasm, plays a crucial role in inflammation diseases and MI repair. Furthermore, our previous study found that FNDC5 pre-conditioning bone marrow-derived mesenchymal stem cells (BMMSCs) could secrete more exosomes, but little was known on MI repair.

**Methods:**

Exosomes isolated from BMMSCs with or without FNDC5-OV were injected into infarcted hearts. Then, cardiomyocytes apoptosis and inflammation responses were detected. Furthermore, exosomes were administrated to RAW264.7 macrophage with LPS treatment to investigate its effect on inflammation and macrophage polarization.

**Results:**

Compared with MSCs-Exo, FNDC5-MSCs-Exo had superior therapeutic effects on anti-inflammation and anti-apoptosis, as well as polarizing M2 macrophage in vivo. Meanwhile, the in vitro results also showed that FNDC5-MSCs-Exo decreased pro-inflammatory secretion and increased anti-inflammatory secretion under LPS stimulation, which partly depressed NF‐κB signaling pathway and upregulated Nrf2/HO-1 Axis.

**Conclusions:**

FNDC5-BMMSCs-derived exosomes play anti-inflammation effects and promote M2 macrophage polarization via NF-κB signaling pathway and Nrf2/HO-1 Axis, which may develop a promising cell-free therapy for MI.

**Supplementary Information:**

The online version contains supplementary material available at 10.1186/s13287-021-02591-4.

## Background

Inflammatory responses play an important role in myocardial infarction (MI) [1]. Macrophage polarization plays an important role in post-infarction inflammation and heart repair [2]. In the early stage of post-infarction, M1 macrophages which secrete pro-inflammatory cytokines, such as tumor necrosis factor-α (TNF‐α), interleukin 6 (IL-6), and interleukin 1β (IL-1β) and high express CD11b and CXCL10 [3], are recruited into infarct myocardium to be responsible for pro-inflammation responses. But, during the later phage of MI, M2 macrophages which are characterized by anti-inflammatory cytokines interleukin 10 (IL-10), with high expression of CD206 and arginase I (Arg 1) [4], are dominant and participate in anti-inflammation effect and cardiac repairment [5]. Thus, to regulate a balance between M1 and M2 macrophages is essential for MI.

Increasing evidences indicate that transplantation of bone marrow-derived mesenchymal stem cells (BMMSCs) serves as a potential therapeutic for MI by anti-inflammatory, reducing fibrosis and enhancing angiogenesis [6]. However, many clinical and animal studies have found that the poor local microenvironment and excessive inflammation responses of ischemic myocardial tissue cause early death of engrafted cells after BMMSCs administration, which limits the therapeutic efficacy of BMMSCs for MI [7, 8]. Recently, exosomes have been found to be vesicles secreted by cells, containing a variety of biological substances, and mediates cell-to-cell communication, as well as participates in various pathophysiological processes such as immune regulation and injured tissue repair [9–11]. Study has shown that stem cell-derived exosomes have the effects of immune regulation, anti-inflammatory, reducing fibrosis, inhibiting oxidative stress and enhancing angiogenesis [12], suggesting they may serve as alternatives to cell therapy of BMMSCs transplantation in MI. However, previous studies have found that exosomes derived by usual stem cells have limitations to repair myocardial tissue; pre-treatment of stem cells can enhance their protective effect on ischemic myocardium [13–15]. This suggests that optimized stem cells can enhance the protective effect of exosomes on ischemic myocardium.

FNDC5, a transmembrane protein that possesses two domains (fibronectin III and carboxy-terminal, respectively) located in the cytoplasm [16], has been proposed that this molecule plays an important role in converting the white adipose tissue to brown adipose tissue and regulating the energy expenditure [17]. Apart from this, its expression and role in various other conditions such as inflammation, cardiovascular diseases, aging and other metabolic conditions have been reported [18–20]. In addition, our previous study showed that FNDC5 pre-treatment BMMSCs improved MI repair [21]. Interestingly, we also found that BMMSCs with overexpression of FNDC5 secreted more exosomes compared ordinary BMMSCs. However, the underlying mechanism is unknown. Therefore, the present study aimed to determine whether exosomes derived from genetically modified BMMSCs overexpressing FNDC5 may improve MI repair by regulation of inflammation.

## Methods

### Animal

Adult male C57BL/6 mice (8–12 weeks of age, 20–25 g) from the PLA Rocket Force Characteristic Medical Center (Beijing, China) were placed into a temperature-controlled animal facility with a 12-h light/ dark cycle (light cycle, 8:00 a.m. to 8:00 p.m.), with tap water and rodent chow provided ad libitum. All animal studies were undertaken in accordance with the regulations and guidelines of Institutional Animal Care and Use Committee of PLA Rocket Force Characteristic Medical Center ((ID: 5034, Beijing, China) and were in compliance with the Guidelines for the Care and Use of Laboratory Animals, as published by the National Academy Press. Completely randomized design and blinding were adopted in animal experiments.

### Isolation, culture and treatments of BMMSCs

BMMSCs were isolated and culture as described previously [22]. Briefly, bone marrow was flushed from the femur and tibia of adult male C57BL/6 mice (8–12 weeks of age, 20–25 g) with Fetal bovine serum (FBS) (Invitrogen, Carlsbad, CA, USA)-free Dulbecco’s modified Eagle’s medium (DMEM) (Corning, Manassas, VA, USA). After passing through a 70-μm strainer and centrifugation at 1200 rpm for 5 min, the cell pellet was re-suspended by DMEM supplemented with 20% FBS and incubated at 37 °C in an atmosphere containing 5% CO_2_ for 24 h. The medium was replaced to remove the non-adherent cells and then was completely replaced every 3 days. Third-generation BMMSCs with optimal growth were applied for further treatments. Furthermore, cells were treated by FNDC5 overexpression as described previously [21].

### Exosome isolation and identification

Cells were cultured with DMEM containing 5% exosome-depleted FBS (Thermo Fisher Scientific). Subsequently, the supernatant was collected and centrifuged at 2000 g for 20 min followed by 10,000 g for 30 min at 4 °C to remove cell debris, apoptotic bodies and microvesicles. Furthermore, the supernatant was filtered with 0.22-μm filters. Isolation of exosomes was performed with ultracentrifugation as described previously [23]. The isolated exosomes were verified by transmission electron microscopy (TEM, Hitachi H7650 TEM). Nanoparticle tracking analysis (NTA) was applied to record the concentration and size distribution of exosomes using NanoSight NS300.

### Induction of MI model and intramyocardial injection of exosomes

Mice MI model was accomplished by ligation of the left anterior descending (LAD) artery [24]. In brief, C57BL/6 mice were anesthetized with isoflurane and mechanically ventilated. The heart was exposed between the fourth and fifth ribs by left thoracotomy. Next, LAD artery was permanently ligated using a 6–0 polyester suture. Success of the ligation was confirmed when the anterior wall of the left ventricle (LV) turned pale and characteristic electrocardiographic (ECG) changes were recorded.

The mice were randomized into four groups: sham-operated group (sham, *n* = 22), MI + PBS group (*n* = 28), MI + MSCs-Exo group (*n* = 26) and MI + FNDC5-MSCs-Exo group (*n* = 28). Immediately after LAD ligation, exosomes suspended in PBS were injected into myocardium at four sites around the infarct border zone [25]. Mice were then killed at 3 and 7 days after MI for tissue harvesting.

### Detection of heart function

Cardiac function was measured as described previously [21]. Briefly, mice were anesthetized (2% isoflurane and oxygen) and put in a supine position. M-mode images and grayscale two-dimensional parasternal short-axis images at the mid-papillary level of each mouse were recorded. Measurements were carried out offline by a single observer in a group-blinded manner. The left ventricular end-systolic diameter (LVESd) and end-diastolic diameter (LVEDd) were measured from M-mode images. Meanwhile, left ventricular end-systolic volume (LVESV) and left ventricular end-diastolic volume (LVEDV) were also measured to calculate left ventricular ejection fraction (LVEF) and fractional shortening (FS) with the following equations: LVEF = (LVEDV − LVESV)/LVEDV × 100% and LVFS = (LVEDd − LVESd)/LVEDd × 100%. All the echocardiographic measurements were performed for three times in a blinded manner.

### Measurement of cardiomyocytes apoptosis

Apoptotic cardiomyocytes in the infarcted heart were detected by a Terminal deoxynucleotidyl transferase dUTP nick‐end labeling (TUNEL) Assay Kit (In Situ Cell Death Detection Kit; Roche Diagnostics) according to the manufacturer's instructions and detected by confocal microscopy (Olympus Fluoview 2000). Furthermore, the total number of nuclei and the number of TUNEL-positive nuclei were determined in five random fields in each sample. The percentage of apoptotic cells was calculated by image J software. All these assays were performed and counted in a blinded manner.

### Enzyme‐linked immunosorbent assay (ELISA)

Mice serum and cell medium supernatants were collected and stored at − 20 °C for the subsequent analysis of cytokines. ELISA kits, including mouse TNF‐α (DKW12‐2720‐096), mouse IL‐6 (DKW12‐2060‐096), mouse IL-10 (BMS614-2; eBioscience) and mouse IL-1β (BMS6002; eBioscience), were performed in accordance with the manufacturer's instructions.

### Hematoxylin and eosin (H&E) staining

Different treatments mice were killed at 3 and 7 days after myocardial injection. Then, the fresh heart was fixed in 4% paraformaldehyde, embedded in paraffin and sectioned at 5-μm intervals. H&E staining of heart sections was performed as described previously [26].

### The treatments of Raw264.7 cells

Raw264.7cells were purchased from American Type Culture Collection. Cells were co-cultured with different concentrations of exosome suspension (5, 10, 15 and 20 μg/mL) for 24 h as previously described [27]. Then, they were cultured with LPS (100 ng/mL) for another 24 h to perform further study. For molecular mechanisms, cells were pre-treated with 20 μM HO-1 inhibitor SnPP for 6 h [28].

### Cell counting kit‐8 (CCK‐8) assay

Cell viability was detected using CCK-8 assay (Dojindo Laboratories Co., Ltd.) according to the manufacturer's instructions. In brief, cells were cultured in 96-well plates in the medium of different treatment groups for 24 h. Then, 10 μL of CCK-8 solution was added to each well and incubated for1.5 h. Finally, the absorbance was measured at 450 nm by using a microtitre plate reader.

### RNA isolation and reverse transcription polymerase chain reaction (RT-PCR) analysis

The total RNAs were extracted with TRIzol reagent following the manufacture’s instruction (Invitrogen, CA, USA) [19]. The cDNAs were prepared using SuperScript III reverse transcriptase and oligo primers (Thermo, Waltham, MA, USA) [29]. The *GAPDH* fragment was amplified as a reference gene. These primers for mouse gene were listed as follows:

- Arg1: forward, 5’-CTCCAAGCCAAAGTCCTTAGAG-3’ reverse, 5’-AGGAGCTGTCATTAGGGACATC-3’.
- CXCL10: forward, 5’-TCTGAGTGGGACTCAAGGGAT-3’ reverse, 5’-TCGTGGCAATGATCTCAACACG-3’.
- GAPDH: forward, 5’-GGGTCCCAGCTTAGGTTCAT-3’ reverse, 5’- CTCGTGGTTCACACCCATCA-3’.

### Immunofluorescence staining

Cells were fixed with 4% paraformaldehyde in PBS for 0.5 h, permeabilized with 0.5% Triton X-100 for 10 min and blocked in 5% normal goat serum in PBS for 1 h at room temperature. Next, cells were probed with anti-Nrf2 antibodies and Alexa Fluor 594-conjugated goat anti-rabbit IgG secondary antibody. After staining the cell nuclei with DAPI for 5 min, the immunofluorescent images were captured on a Zeiss fluorescence microscopy (Jena, Germany). For immunofluorescence co‐staining, 49,6-diamidino-2-phenylindole (DAPI) (Sigma) stained all cell nuclei. Additional staining was performed with a monoclonal antibody against Troponin I (cTnI, Santa Cruz) and CD206 (Abcam) for the identification of myocardium and macrophages, respectively. Sections were imaged using confocal microscope (Fluo-View-FV1000, Olympus, Japan).

### Western blot assay

Western blot analysis was performed as previously described [21]. Here, cells were collected, normalized and separated by 12% sodium dodecyl sulphate–polyacrylamide gel electrophoresis (SDS-PAGE) and transferred to polyvinylidene difluoride (PVDF) membranes (EMD Millipore). After 1 h of blocking with 5% non‐fat milk in 1xTBST at room temperature, the membranes were incubated with primary antibodies (1:500–1:1000) overnight at 4 °C and then were incubated with secondary antibody (1:5000) for 1 h at room temperature. Eventually, the membranes were washed three times with 1xTBST and detected by enhanced chemiluminescence (ECL) system (Amersham Bioscience). Densitometric analysis of Western blot was determined with VisionWorks LS, version 6.7.1.

### Statistics analysis

Data analysis was performed using GraphPad Prism 5.0 (San Diego, CA, USA). All data in our study are presented as the mean ± SEM. Shapiro–Wilk normality test was performed to evaluate the normality of the data distribution. Comparisons of parameters among three or more groups were performed with a one-way analysis of variance (ANOVA). Differences between 2 groups were compared by using Student’s *t* test. The Bonferroni testing was performed to determine the post hoc testing. *P* value < 0.05 was considered statistically significant.

## Results

### Intramyocardial injection of exosomes derived from FNDC5-MSCs significantly attenuated cardiomyocyte apoptosis and improved short-term cardiac function in MI mice

**Fig. 1:**
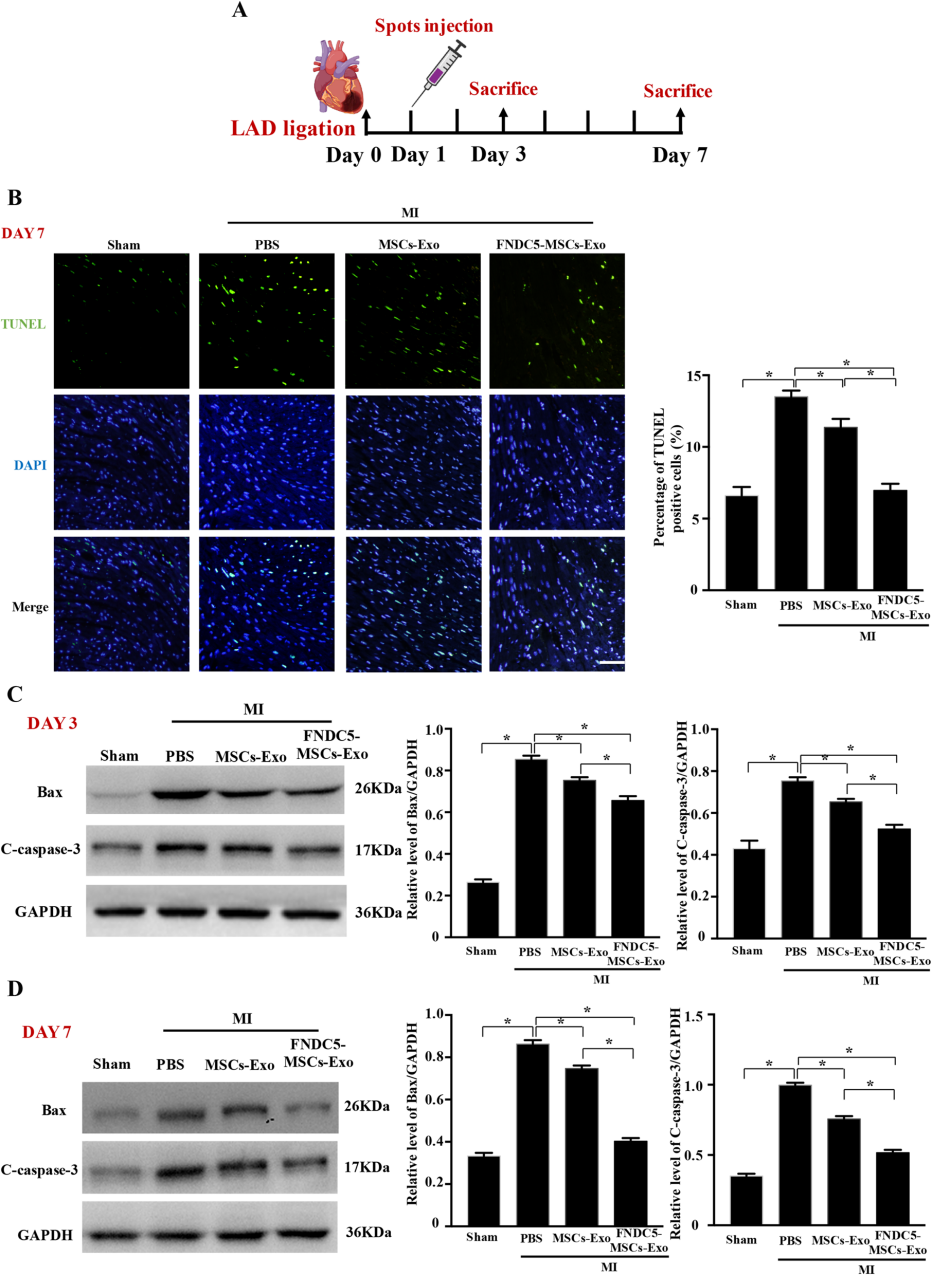
Intramyocardial spots injection of exosomes derived from FNDC5-MSCs significantly suppressed cardiomyocyte apoptosis in MI mice. **a** The diagrammatic sketch of MI induction and exosome intramyocardial injection and mice were killed at 3 and 7 days after intramyocardial injection (*n* ≥ 14). **b** The apoptosis of cardiomyocytes at 7 days was assessed by a TUNEL Assay Kit (scale bars = 20 μm). Nucleus was blue with DAPI and the TUNEL-positive apoptotic cardiomyocytes were green. The average fluorescence intensity of positive apoptotic cardiomyocytes was quantified. The protein of Bax and cleaved caspase‐3 within the heart tissue at 3 days (**c**) and 7 days (**d**) was detected by Western blot and semi-quantification analysis. Data are expressed as the means ± SEM; *n* = 5; *P < 0.05

Exosomes were isolated by density-gradient ultracentrifugation. As shown in Additional file 1: Figure S1A, B, exosomes showed a cup‐shaped morphology by TEM, and the size of exosomes was about 100 nm by NTA. Meanwhile, markers of exosomes (CD63, CD81 and ALIX) were highly expressed in FNDC5-MSCs‐Exo groups compared with MSCs-Exo group (Additional file 1: Figure S1C). 50 μg MSCs-Exo or FNDC5-MSCs-Exo dissolved in 50 μL PBS was injected into the infarct border zone at four spots (Fig. 1a).

TUNEL assay was used to detect the cardiomyocyte apoptosis after MI. As shown in Fig. 1b, TUNEL-positive (green) cardiomyocytes in MSCs-Exo and FNDC5-MSCs-Exo group were decreased compared with PBS group. Meanwhile, the quantitative analysis reveals that the percentages of TUNEL-positive cells exposed to MSCs-Exo and FNDC5-MSCs-Exo group were 11.37 ± 0.59% and 6.97 ± 0.46%, respectively, significantly decreased as opposed to the PBS group (13.5 ± 0.43%, *P* < 0.05). What is more, compared with MSCs-Exo group, the apoptosis rate of cells was remarkably decreased in FNDC5-MSCs-Exo group (*P* < 0.05). Western blot analysis further confirmed the cardioprotective effect of MSCs-Exo and FNDC5-MSCs-Exo with reducing protein amounts of Bax and cleaved caspase‐3 in ischemic heart tissue (Fig. 1c, d, *P* < 0.05). Taken together, FNDC5-MSCs-Exo could attenuate cardiomyocyte apoptosis after MI.

What is more, we followed up for 28 days to detect the heart function by echocardiography. Echocardiographic analysis revealed that the baseline parameters were similar in all groups. However, the LVEDd and LVESd were increased after MI. Meanwhile, the LV dimensions were decreased while the LVEF and FS were increased in the MI + FNDC5-MSCs-Exo group compared with the MI + PBS and MI + MSCs-Exo groups (Additional file 2: Figure S2, *P* < 0.05). Taken together, these data suggested that FNDC5-MSCs-Exo injection had some therapeutic benefits in improving short-term cardiac dysfunction.

### FNDC5-MSCs‐exo administration reduced post‐infarction inflammation and increased M2 macrophage polarization in vivo

**Fig. 2:**
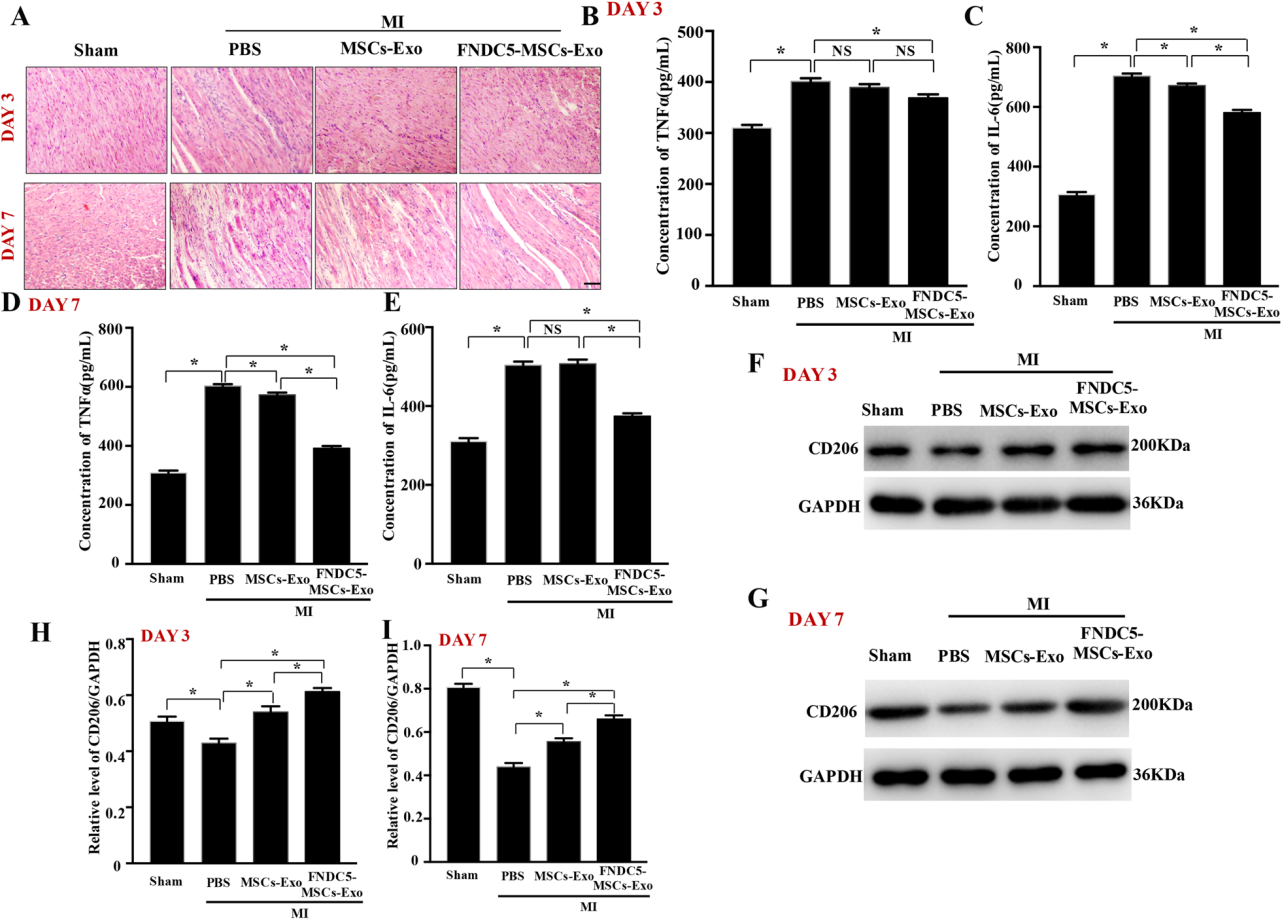
FNDC5-MSCs‐Exo administration reduced post‐infarction inflammation and increased M2 macrophage polarization in vivo. **a** Representative images of inflammatory cells infiltration within the infarct border zone at 3 and 7 days were illustrated by H&E staining. Serum inflammatory cytokines (TNF‐α and IL-6) at 3 days (**b**, **c**) and 7 days (**d**, **e**) were assayed by ELISA. Western blot analysis of CD206 protein at 3 days (**f**) and 7 days (**g**). Representative semi-quantification analysis of CD206 at 3 days (**h**) and 7 days (**i**). Data are expressed as the means ± SEM; n = 5; *P < 0.05

MI injury induced post-infarction inflammation, and a great amount of inflammatory cells assembled and infiltrated into the infarction area. H&E staining showed that in both MSCs-Exo and FNDC5-MSCs-Exo groups, FNDC5-MSCs-Exo obviously alleviated the infiltration of inflammatory cells to protect myocardium from further damage (Fig. 2a). Furthermore, the pro‐inflammatory cytokines, TNF‐α and IL-6, were detected in serum at 3 and 7 days. Compared with MSCs-Exo group, the secretions of TNF‐α and IL-6 were significantly reduced in FNDC5-MSCs-Exo group (Fig. 2b–e, *P* < 0.05).

Macrophage polarization played an essential role in inflammation. Thus, we explored the subsets of macrophages accumulating in the ischemic heart. As shown by representative immunofluorescence co‐staining demonstrated, after quantifying the relative fluorescence intensity of CD206, we discovered that FNDC5-MSCs-Exo effectively increased the expression of CD206 compared with MSCs-Exo group and PBS group (Additional file 3: Figure S3, *P* < 0.05). Western blot analysis further verified that the protein expression of CD206 (M2 marker) in ischemic heart tissue was elevated under MSCs-Exo and FNDC5-MSCs-Exo group (Fig. 2f, g). Furthermore, FNDC5-MSCs-Exo presented better effects than MSCs-Exo on facilitating M2 macrophage polarization after MI (Fig. 2h, i, *P* < 0.05). Collectively, compared with MSCs-Exo, FNDC5-MSCs-Exo reduced post‐infarction inflammation and promoted M2 macrophage polarization in mice.

### FNDC5-MSCs‐exo ameliorates inflammation responses by increasing anti‐inflammatory cytokines, as well as decreasing pro‐inflammatory cytokines in Raw264.7 cells

**Fig. 3:**
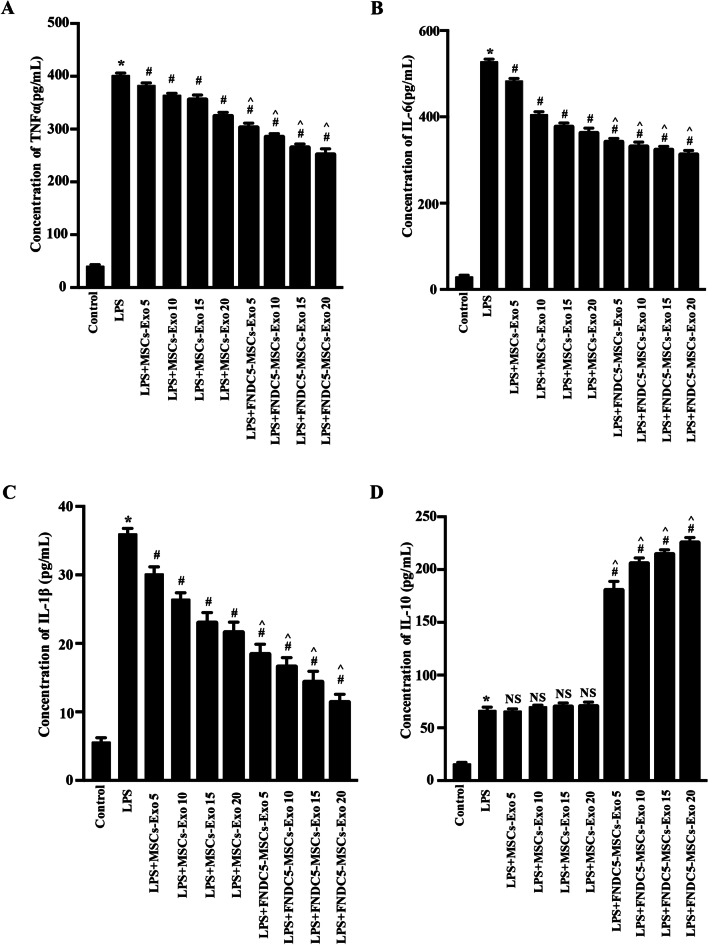
FNDC5-MSCs‐Exo ameliorates inflammation responses by increasing anti‐inflammatory cytokines, as well as decreasing pro‐inflammatory cytokines in Raw264.7 cells. The secretions of pro‐inflammatory cytokines such as TNF‐α (**a**), IL‐6 (**b**) and IL-1β (**c**) and anti-inflammatory cytokine such as IL-10 (**d**) secreted from Raw264.7 cells were detected by ELISA. Data are expressed as the means ± SEM; n = 5; *P < 0.05 versus control group; ^*#*^*P* < 0.05 versus LPS group; ^*^*^*P* < 0.05 versus MSCs-Exo group under equal concentration

To further investigate the effects of MSCs-Exo, FNDC5-MSCs-Exo on inflammation responses, exosomes were administrated to Raw264.7 cells under LPS treatment (100 ng/mL). To optimize the reasonable dose of exosomes, we detected the effects of different doses of exosomes (5, 10, 15 and 20 μg/mL) on inflammatory cytokines. ELISA analysis showed that the pro‐inflammatory cytokines (IL‐6, TNF‐α and IL-1β) were decreased while the anti-inflammatory cytokine (IL-10) was increased in both Exo and L-Exo groups by a dose-dependent manner (Fig. 3a–d, *P* < 0.05). Moreover, the pro‐inflammatory cytokines were dramatically decreased by exosomes at 20 μg/mL (*P* < 0.05). Thus, the dose of exosomes was performed to further study.

CCK-8 assay was performed to confirm that MSCs-Exo and FNDC5-MSCs-Exo reduced pro-inflammatory cytokines without affecting cell viability. Data showed no significant influence on cell viability (Additional file 4: Figure S4).

In summary, our data indicated that FNDC5-MSCs‐Exo had superior therapeutic effects on anti-inflammation by increasing anti‐inflammatory cytokines and decreasing pro‐inflammatory cytokines.

### FNDC5-MSCs‐exo dramatically increased M2 macrophage polarization and decrease M1 macrophage polarization in vitro

**Fig. 4:**
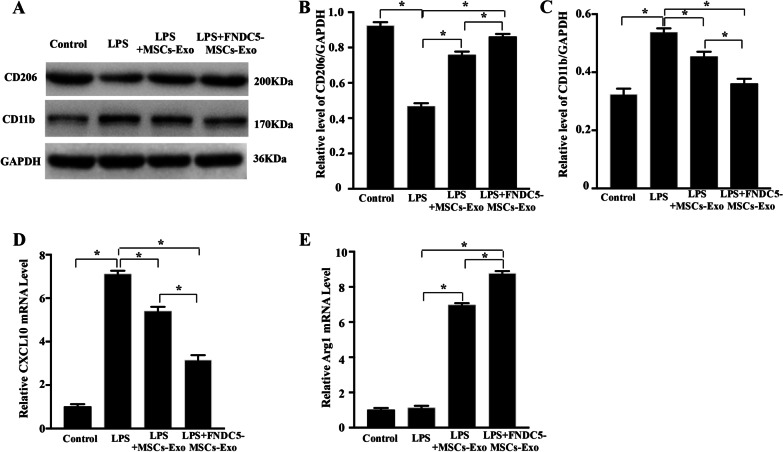
FNDC5-MSCs‐Exo dramatically increased M2 macrophage polarization and decrease M1 macrophage polarization in vitro. **a** Western blot analysis of CD206 and CD11b in RAW264.7 cells with different treatments. Representative semi-quantification analysis of CD206 (**b**) and CD11b (**c**). Representative relative mRNA level of CXCL10 (**d**) and Arg 1 (**e**). Data are expressed as the means ± SEM; *n* = 5; *P < 0.05

To explore macrophage polarization, we detected macrophages markers CD11b, CXCL10 (M1 macrophages) and CD206, ArgI (M2 macrophages). Western blot analysis indicated that MSCs-Exo, FNDC5-MSCs-Exo significantly increased the expression level of CD206, while markedly reduced the expression level of CD11b compared with LPS group (Fig. 4a–c, *P* < 0.05). Furthermore, the RT‐qPCR results showed that the mRNA level of CXCL10 remarkably reduced and Arg1 was significantly elevated in FNDC5-MSCs-Exo group (Fig. 4d–e, *P* < 0.05).

Taken together, these results showed that FNDC5-MSCs‐Exo markedly promoted M2 macrophage polarization while reduced M1 macrophage polarization.

### FNDC5-MSCs‐exo reduced the inflammation by suppressing the NF‐κB signaling pathway and upregulating Nrf2/HO-1 Axis

**Fig. 5:**
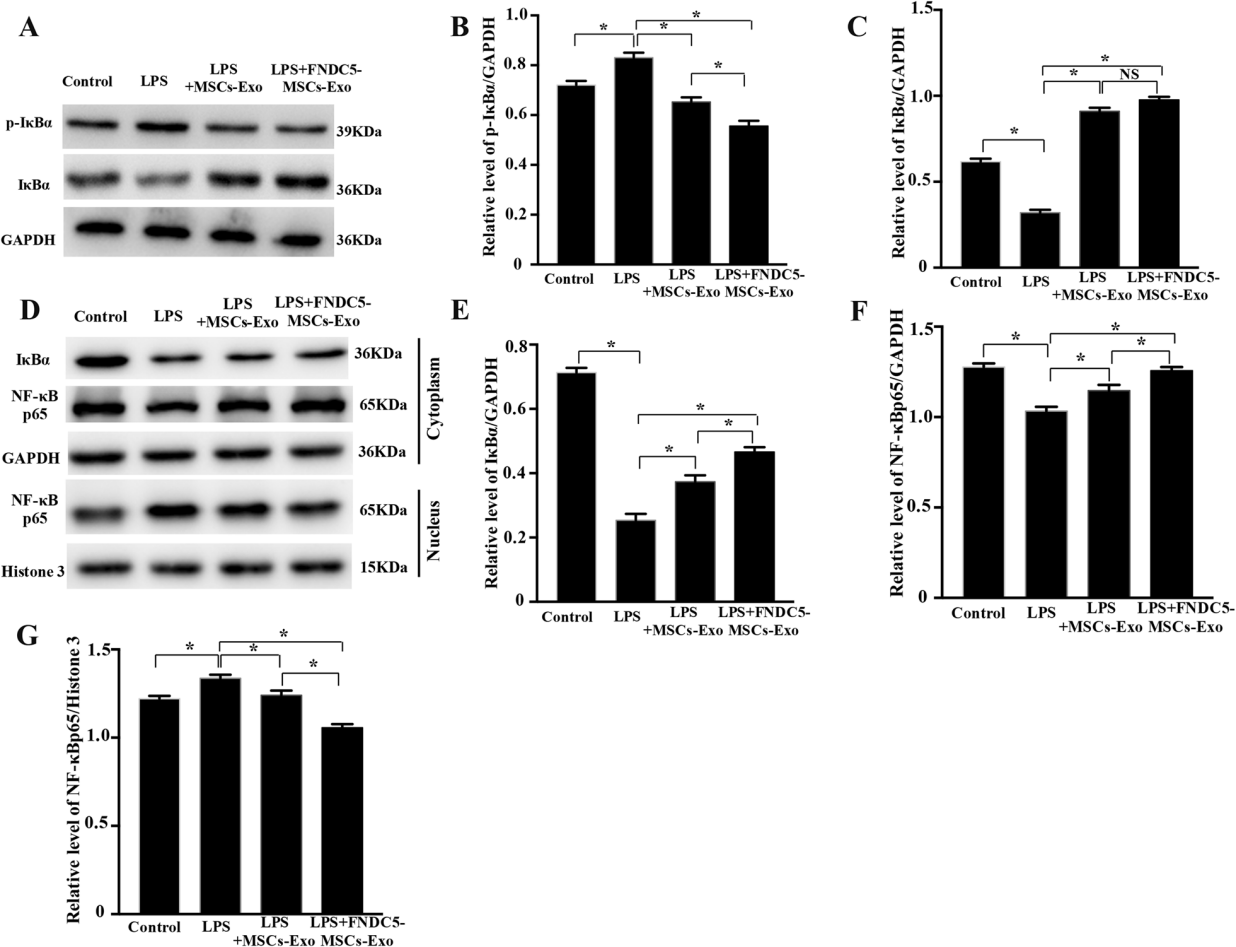
FNDC5-MSCs‐Exo reduced the inflammation by suppressing the NF‐κB signaling pathway. **a** Representative Western blot analysis of p-IκBα and IκBα in RAW264.7 cells under different conditions. Representative semi-quantification analysis of p-IκBα (**b**) and IκBα (**c**). **d** Cytoplasmic and nuclear proteins were collected and extracted to measure IκBα and NF‐κB p65. GAPDH (**e**, **f**) and Histone3 (**g**) were used as normalized cytoplasmic and nuclear protein. Data are expressed as the means ± SEM; *n* = 5; *P < 0.05.

**Fig. 6:**
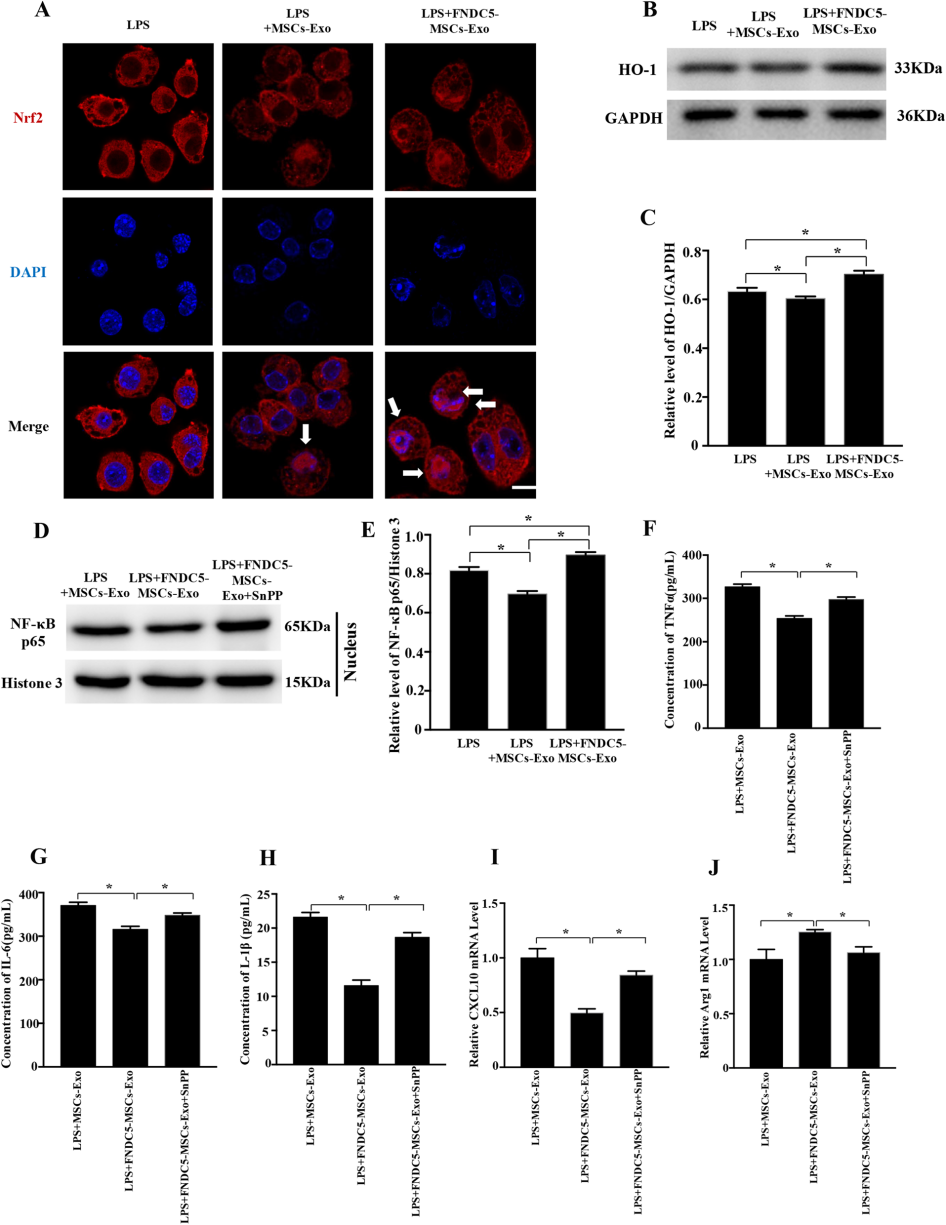
FNDC5-MSCs‐Exo reduced the inflammation by upregulating Nrf2/HO-1 Axis. **a** Representative Immunostaining of intracellular Nrf2 (scale bars = 20 μm). After 24 h treatment with LPS, the intracellular Nrf2 was detected by immunostaining with specific antibody, whereas the nuclei were detected by DAPI staining. **b** Western blot analysis of HO-1 under different conditions. **c** Representative semi-quantification analysis of HO-1. **d** Nuclear proteins were collected and extracted to measure NF‐κB p65. **e** Histone3 were used as normalized cytoplasmic and nuclear protein. The secretions of TNF‐α (**f**), IL‐6 (**g**) and IL-1β (**h**) were detected by ELISA. Representative relative mRNA level of CXCL10 (**i**) and Arg 1 (**j**). Data are expressed as the means ± SEM; *n* = 5; *P < 0.05

To further understand the molecular mechanism of inflammation regulated by exosomes, we evaluated the effects of MSCs‐Exo and FNDC5-MSCs‐Exo on LPS‐dependent NF‐κB signaling pathway. The Western blot results showed that compared with LPS group, total protein of IκBα was increased while p-IκBα was decreased in both MSCs‐Exo and FNDC5-MSCs‐Exo group (Fig. 5a–c, *P* < 0.05). Furthermore, NF‐κB p65 translocation from the cytoplasm to nucleus was suppressed (Fig. 5d–e, *P* < 0.05). We also found that compared with MSCs-Exo group, FNDC5-MSCs‐Exo prominently suppressed the nuclear translocation of NF‐κB p65 (Fig. 5f, g, *P* < 0.05). Meanwhile, we focused on the effects of MSCs‐Exo and FNDC5-MSCs‐Exo on the Nrf2/HO-1 pathway by immunostaining and Western blot analysis. As indicated in Fig. 6a, FNDC5-MSCs‐Exo obviously induced the nuclear translocation of Nrf2. Then, Western blot analysis also suggested that FNDC5-MSCs‐Exo increased HO-1 expression (Fig. 6b, c, *P* < 0.05).

To clarify the impact of HO-1 on the anti-inflammatory activities of FNDC5-MSCs‐Exo, HO-1 specific inhibitor Sn (IV) protoporphyrin IX dichloride (SnPP, 20 μM) was administrated to block the enzyme activity of HO-1(Additional file 5: Figure S5A, B *P* < 0.05). As shown in Fig. 6d, e, SnPP attenuated the inhibitory effect of FNDC5-MSCs‐Exo on nucleus translocation of NF-ĸB p65 (*P* < 0 0.05). ELISA analysis suggested that HO-1 inhibitor dramatically recovered secretion of pro-inflammatory cytokines such as TNF‐α, IL‐6 and IL-1β (Fig. 6f–h, *P* < 0.05). Furthermore, the RT‐qPCR results showed that HO-1 inhibitor significantly elevated the mRNA level of CXCL10 while reduced that of Arg1 compared with FNDC5-MSCs-Exo group (Fig. 6i, j, *P* < 0.05).

**Fig. 7:**
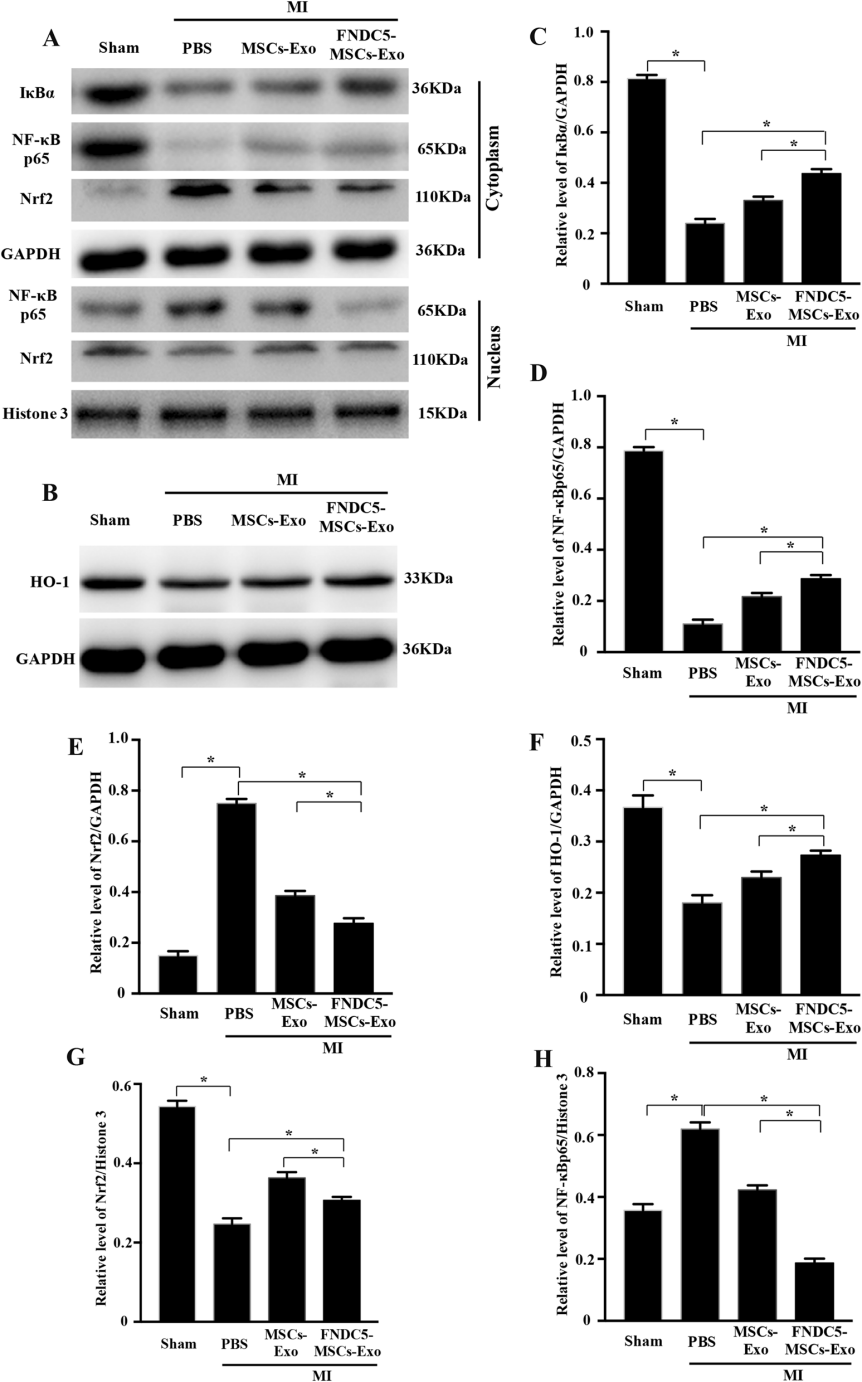
Molecular mechanisms proteins in vivo. **a**, **b** Representative Western blot analysis of IκBα and NF‐κB p65, Nrf3 in heart tissue under different conditions. Cytoplasmic and nuclear proteins were collected and extracted to measure IκBα and NF‐κB p65, Nrf3, GAPDH (**c**–**f**) and Histone3 (**g**, **h**) were used as normalized cytoplasmic and nuclear protein. Data are expressed as the means ± SEM; *n* = 5; *P < 0.05

To ensure the molecular mechanism of inflammation regulated by exosomes i*n vivo*, the molecular mechanisms proteins of heart tissue were detected. Our results indicated that total protein of IκBα was increased in both MI + MSCs‐Exo and MI + FNDC5-MSCs‐Exo group (Fig. 7a, c, *P* < 0.05). Furthermore, NF‐κB p65 translocation from the cytoplasm to nucleus was suppressed (Fig. 7a, d, h, *P* < 0.05). Meanwhile, we also found that compared with MSCs-Exo group, FNDC5-MSCs‐Exo prominently suppressed the nuclear translocation of NF‐κB p65. In addition, as indicated in Fig. 7b, e–g, FNDC5-MSCs‐Exo obviously induced the nuclear translocation of Nrf2 (*P* < 0 0.05) and increased HO-1 expression (*P* < 0 0.05). Thus, we demonstrated that FNDC5-MSCs‐Exo reduced the inflammation by suppressing the NF‐κB signaling pathway and upregulating Nrf2/HO-1 Axis.

## Discussion

**Fig. 8:**
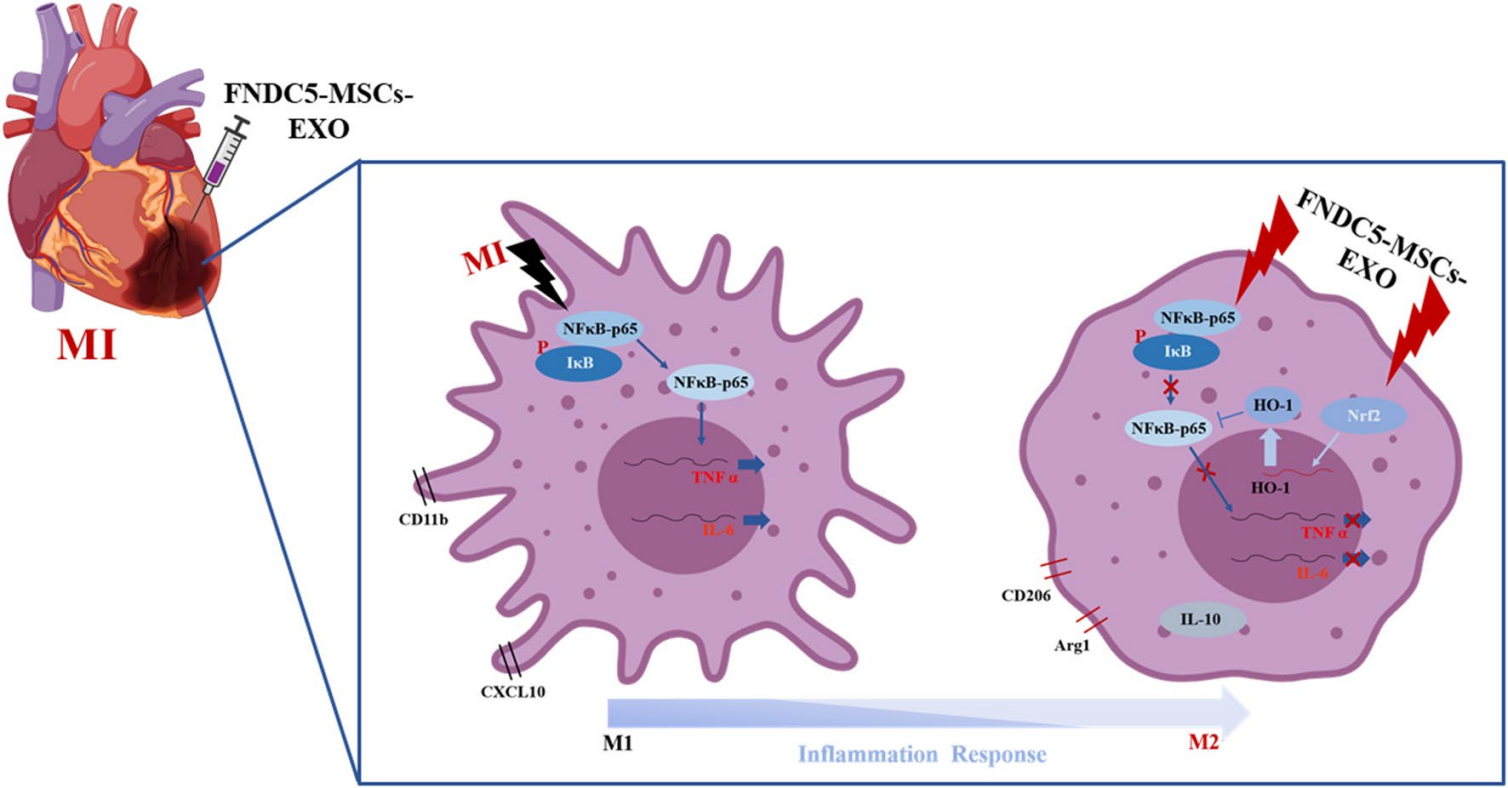
Mechanism of FNDC5-MSCs‐Exo improves myocardial injury. FNDC5-MSCs‐Exo reduces inflammation and promotes M2 macrophage polarization via down-regulating NF-κB signaling pathway and up-regulating Nrf2/HO-1 Axis

Our previous study found that FNDC5 pre-treatment improved BMMSCs therapy effect for MI [21]. Interestingly, we also found that FNDC5-optimized BMMSCs secreted more exosomes. However, its role in post-infarction inflammation remains unclear. Therefore, the present study aimed to evaluate the effect of exosomes derived from optimized BMMSCs on inflammation after MI injury. Here, our study led to two key findings as follows: (1) FNDC5-MSCs-Exo had superior therapeutic effects on anti-inflammation and anti-apoptosis, as well as polarizing M2 macrophage. (2) FNDC5-MSCs-Exo decreased the secretion of pro-inflammatory cytokines in macrophage, while increased the anti-inflammatory cytokine under LPS stimulation. Collectively, our data suggested that FNDC5-MSCs-Exo may be an optimizing strategy of BMMSCs-based therapy for MI (Fig. 8).

Although transplantation of BMMSCs serves as a potential therapeutic for MI by paracrine effects, many clinical and animal studies have found that the poor local microenvironment and excessive inflammation responses of ischemic myocardial tissue cause early death of engrafted cells after usual BMMSCs administration, which limits the therapeutic efficacy of BMMSCs for MI [7, 8]. Our and other previous studies focused on improvement in the low translated cells survival by optimizing BMMSCs [21, 30, 31]. However, the specific mechanisms were unclear. Previous studies have shown that exosomes secreted by stem cells play multiple effects, including immune regulation, anti-inflammatory, anti-apoptosis, reducing fibrosis, inhibiting oxidative stress and enhancing angiogenesis [12, 32], suggesting exosomes may be alternatives to cell therapy for MI. However, increasing studies have found that exosomes derived by usual stem cells have limitations to repair myocardial tissue; pre-treatment of stem cells can enhance their protective effect on ischemic myocardium [13–15]. In the present study, we discovered that compared with MSCs-Exo, FNDC5-MSCs-Exo could significantly ameliorate post-infarction short-term heart function and cardiomyocytes apoptosis. It suggested that FNDC5-MSCs-Exo contributed to relieving myocardial injury and ameliorating cardiac function after MI. Therefore, further studies need to be conducted to concerning the effect of exosomes on long-term cardiac function by expand of sample size.

Inflammation responses have an essential effect during MI [33]. After MI, macrophages were recruited into the ischemic myocardial tissue and participate in inflammation responses and myocardial repair [34, 35], suggesting the key of post-infarction repair on anti-inflammation and remaining the balance of macrophage polarization. In the present study, MSCs-Exo and FNDC5-MSCs-Exo reduced the infiltration of inflammatory cells and improved secretion of pro-inflammatory cytokines in the ischemic heart tissue. Meanwhile, we also found that FNDC5-MSCs-Exo had more effective influence on M2 macrophage polarization in vivo. To further investigate the effect of exosomes on inflammation, we co-cultured Raw264.7 cells with different exosomes after LPS treatment. Our data indicated that MSCs-Exo and FNDC5-MSCs-Exo increased anti‐inflammatory cytokines (IL-10) and decreased pro‐inflammatory cytokines (IL‐6, TNF‐α and IL-1β), as well as increased M2 macrophage marker Arg1, and decrease M1 macrophage marker CXCL10, suggesting MSCs-Exo and FNDC5-MSCs-Exo promoted M2 macrophage polarization while suppressed M1 macrophage polarization.

To elucidate the underlying mechanisms, we firstly investigated the effects of MSCs-Exo and FNDC5-MSCs-Exo on the nuclear translocation of NF-κB. As we known, LPS stimuli are known to activate the NF-κB pathway for regulating the expression of immunomodulating and inflammatory genes [36]. NF-κB activation is a complex process involving rapid phosphorylation and degradation of IκB. Ultimately, NF-κB p65 subunits are translocated to the cell nucleus and induce IL‐6 and TNF‐α [37]. Here, Western blotting analysis showed that MSCs-Exo and FNDC5-MSCs-Exo reduced the nuclear translocation of NF-κB in RAW264.7 cells after LPS stimulation. These results suggested that inhibition of NF-κB pathway is the mechanisms by which MSCs-Exo and FNDC5-MSCs-Exo selectively suppresses the expression of pro-inflammatory cytokines. Nrf2 is a transcriptional factor and regulates the cellular defense against oxidative stress [38]. Some anti-inflammatory factors disrupt Keap1-Nrf2 complex, promote the nuclear translocation of Nrf2 and finally activate the transcription of antioxidant genes, such as heme oxygenase-1 (HO-1) [39]. HO-1 is well known to orchestrate the cross-talks between Nrf2 and NF-κB pathways [40]. Therefore, to further elucidate the underlying mechanisms, we focused on the Nrf2/HO-1 pathway. In the present study, we found that FNDC5-MSCs‐Exo strongly induced the nuclear translocation of Nrf2 and increased expression of HO-1protein. Thus, we hypothesized that FNDC5-MSCs‐Exo might regulate inflammation responses and macrophage M2 polarization via activating Nrf2/HO-1 pathway. We further gave cells treatments of HO-1 inhibitor SnPP. Surprisingly, SnPP diminished the inhibitory effect of FNDC5-MSCs‐Exo on NF-κB activation and reducing secretion of pro-inflammatory cytokines in macrophages. Meanwhile, SnPP reversed the effect of FNDC5-MSCs‐Exo on downregulation of M1 macrophage polarization and upregulation of M2 macrophage polarization. These results suggested that FNDC5-MSCs‐Exo activated Nrf2/HO-1 which was the key mechanism for inhibiting NF-κB pathway and subsequently down-regulating LPS-induced inflammation responses.

The present study bears some clinical relevance, but there are limitations. Firstly, we did not follow up MI mice for longer time to observe the changes in cardiac function. Secondly, the detailed molecular mechanism by which exosomes exerted protective effects on inflammation was not clarified completely. Therefore, further research needs to be conducted.

## Conclusions

Thus, our study in vitro and in vivo showed that FNDC5-MSCs-Exo had superior therapeutic effects on anti-inflammation and polarizing M2 macrophage, which partly depressed NF‐κB pathway and upregulated Nrf2/HO-1 Axis. These findings suggested that exosomes from optimized BMMSCs may be a potential target for MI.

## Supplementary Information

**Additional file 1. Figure S1: **Identification of exosomes. **A** MSCs-Exo morphology was observed by TEM at × 50,000 (scale bars =100 nm). **B** Size distribution of exosomes measured in triplicate by NTA. **C** Western blot of exosomes associated markers including CD63, CD81 and ALIX.

**Additional file 2. Figure S2: **Evaluation of heart function after different groups. Histograms illustrating the heart function parameters: left ventricular end diastolic diameter (LVEDd, **A**), left ventricular end systolic diameter (LVESd, **B**), left ventricular ejection fraction (**C**) and left ventricular fractional shortening (**D**). Data are expressed as means ± SEM; *n* = 8; *p < 0.05

**Additional file 3. Figure S3: **Effects of FNDC5-MSCs on the expression of CD206. **A**Representative images of immunofluorescence co staining. DAPI (blue), CD206 (green). **B** The average fluorescence intensity of CD206 was quantified. Data are expressed as means ± SEM; n =5; *p < 0.05

**Additional file 4. Figure S4: **Cell viability of Raw264.7 cells. Representative Cell viability under various treatments. Data are expressed as the means ± SEM; *n* = 5; NS *p* > 0.05

**Additional file 5. Figure S5: **Expression of HO-1 of Raw264.7 cells with SnPP treatment. **A** Western blot analysis of HO-1 under different conditions. **B** Representative semi quantification analysis of HO-1. Data are expressed as the means ± SEM; *n* = 5; *p < 0.05

## Data Availability

The data sets supporting the results of this article are included within the article and its additional files.

## Abbreviations

FNDC5: Fibronectin type III domain-containing protein 5
BMMSCs: Bone marrow mesenchymal stem cells
MI: Myocardial infarction
DMEM: Dulbecco’s modified Eagle’s medium
FBS: Fetal bovine serum
ELISA: Enzyme-linked immunosorbent assay
LAD: Left anterior descending
LV: Left ventricle
ECG: Electrocardiography
TUNEL: Terminal-deoxynucleotidyl transferase mediated-dUTP nick-end labeling
DAPI: 4,6-Diamidino-2-phenylindole
PBS: Phosphate-buffered saline
NTA: Nanoparticle tracking analysis
TEM: Transmission electron microscope
CCK-8: Cell counting Kit-8
TNF‐α: Tumor necrosis factor-α
IL-6: Interleukin
IL-1β: Interleukin 1β
IL-10: Interleukin
Arg 1: Arginase I
HO-1: Heme oxygenase-1
