# Supplementary figures and images for: Exosomes secreted by FNDC5-BMMSCs protect myocardial infarction by anti-inflammation and macrophage polarization via NF-κB signaling pathway and Nrf2/HO-1 axis
Source: Stem Cell Res Ther. 2021 Sep 28;12:519. doi: 10.1186/s13287-021-02591-4 (PMC8480009; doi:10.1186/s13287-021-02591-4)

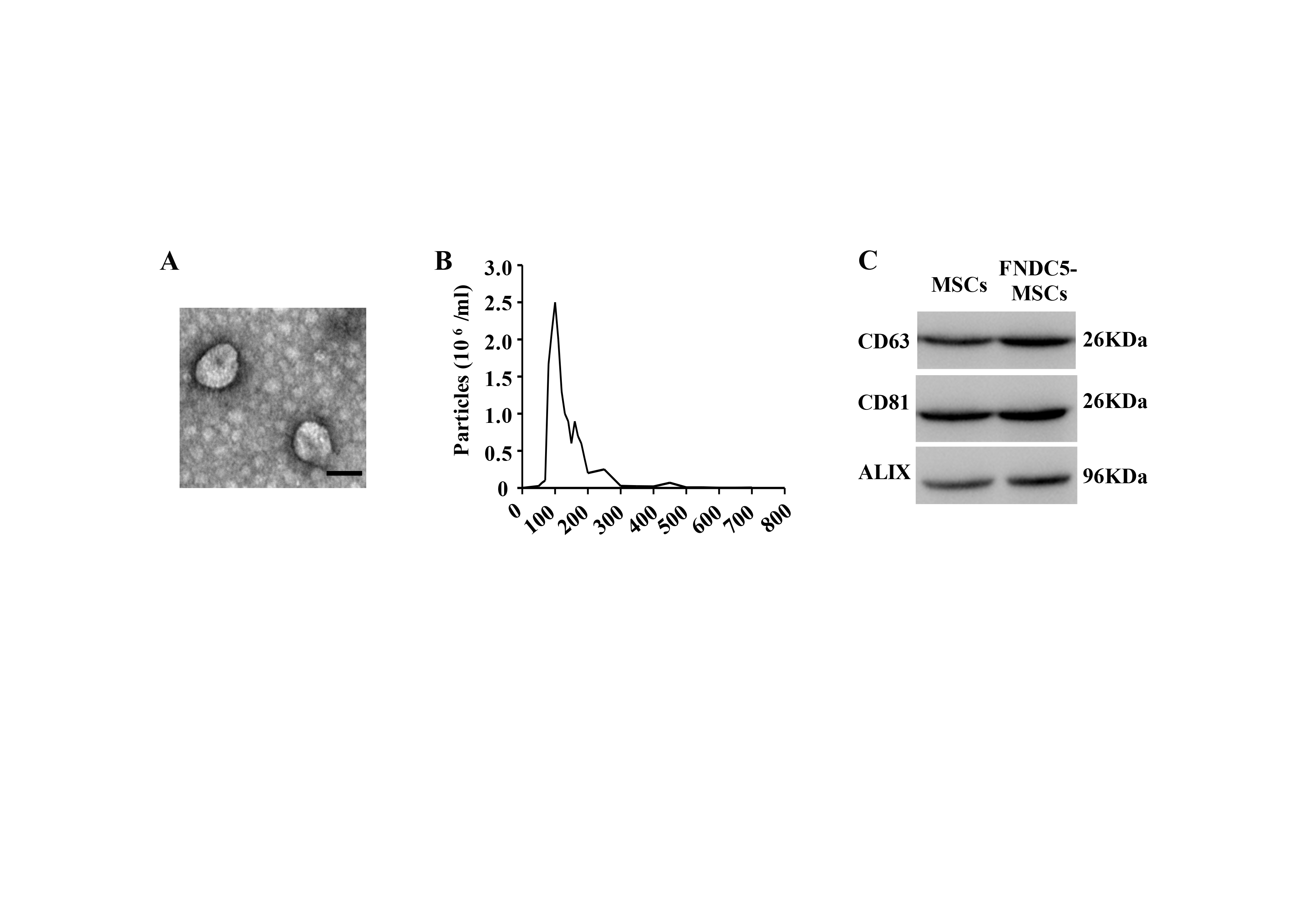

Supplement: Supplementary file 1 — Additional file 1. Figure S1: Identification of exosomes. A MSCs-Exo morphology was observed by TEM at × 50,000 (scale bars =100 nm). B Size distribution of exosomes measured in triplicate by NTA. C Western blot of exosomes associated markers including CD63, CD81 and ALIX. [file 13287_2021_2591_MOESM1_ESM.tif]

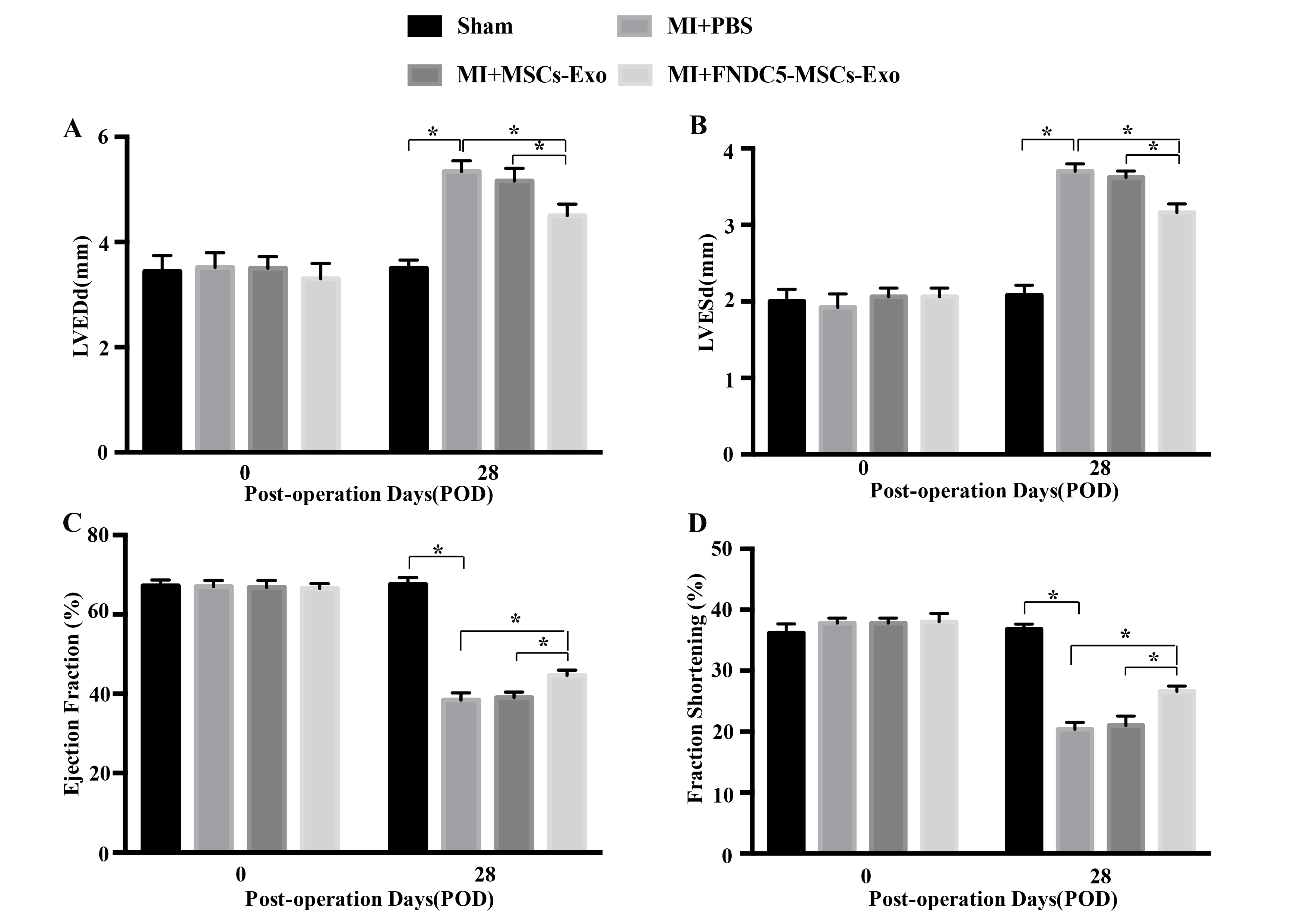

Supplement: Supplementary file 2 — Additional file 2. Figure S2: Evaluation of heart function after different groups. Histograms illustrating the heart function parameters: left ventricular end diastolic diameter (LVEDd, A), left ventricular end systolic diameter (LVESd, B), left ventricular ejection fraction (C) and left ventricular fractional shortening (D). Data are expressed as means ± SEM; n = 8; *p < 0.05 [file 13287_2021_2591_MOESM2_ESM.tif]

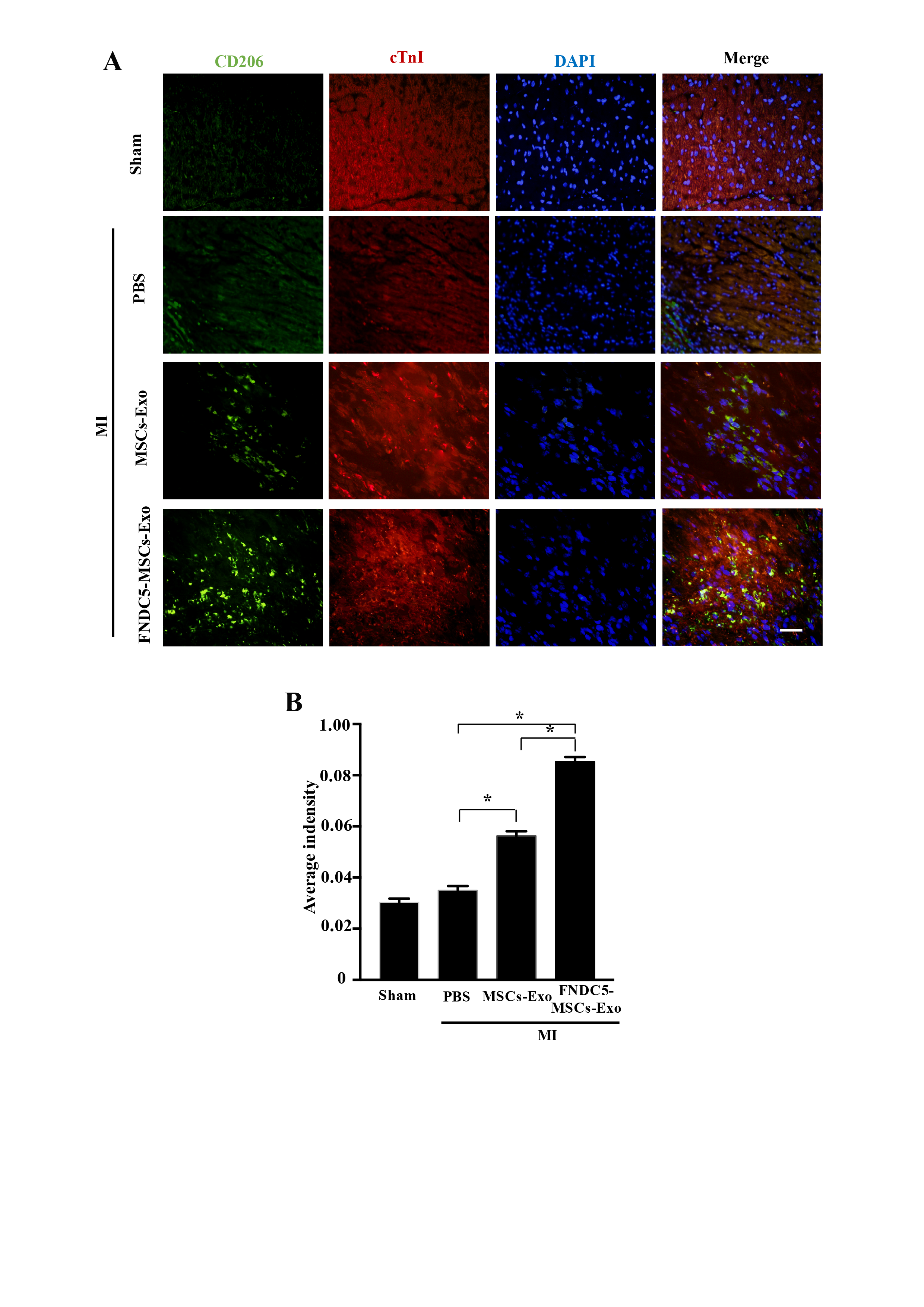

Supplement: Supplementary file 3 — Additional file 3. Figure S3: Effects of FNDC5-MSCs on the expression of CD206. ARepresentative images of immunofluorescence co staining. DAPI (blue), CD206 (green). B The average fluorescence intensity of CD206 was quantified. Data are expressed as means ± SEM; n =5; *p < 0.05 [file 13287_2021_2591_MOESM3_ESM.tif]

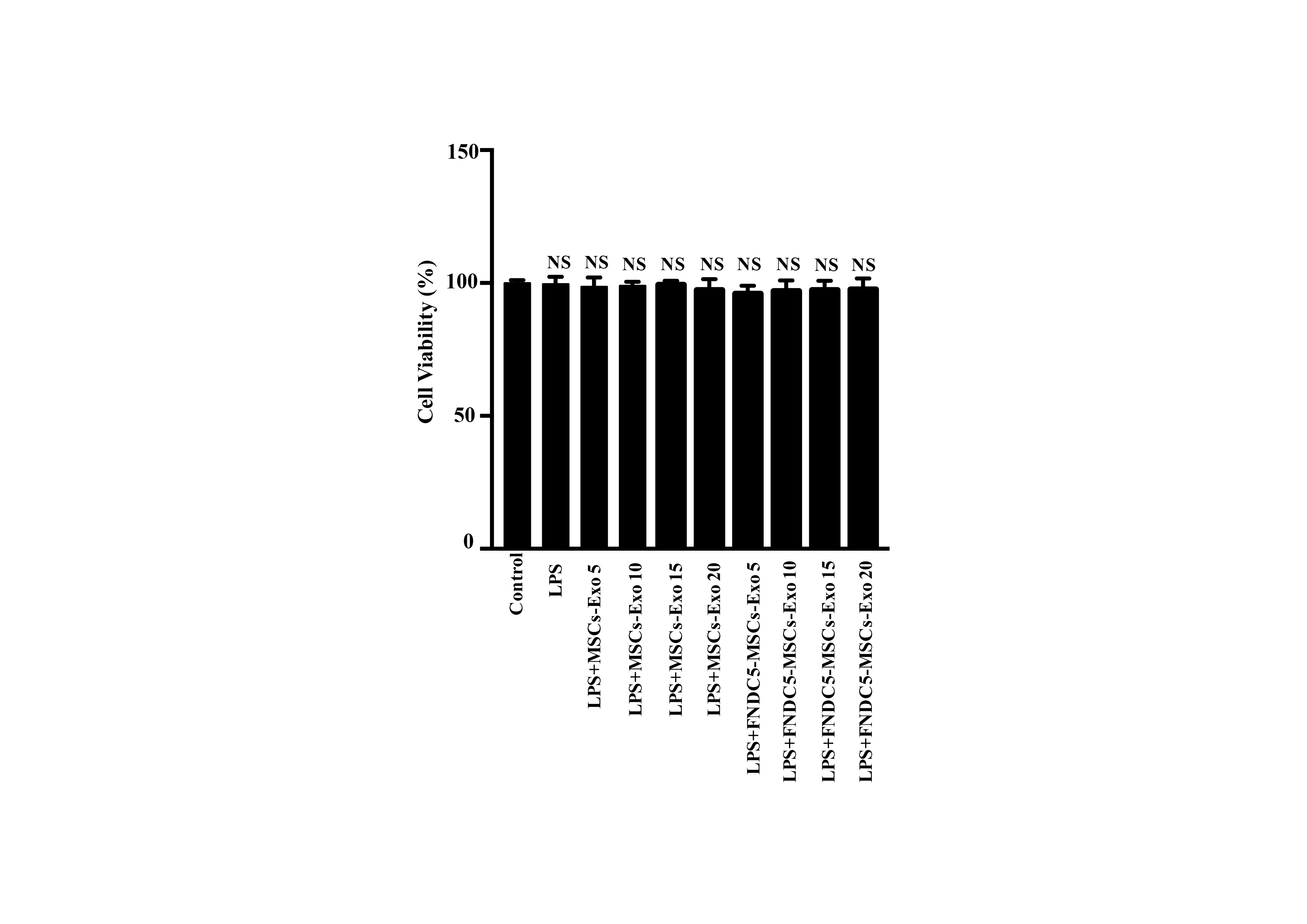

Supplement: Supplementary file 4 — Additional file 4. Figure S4: Cell viability of Raw264.7 cells. Representative Cell viability under various treatments. Data are expressed as the means ± SEM; n = 5; NS p > 0.05 [file 13287_2021_2591_MOESM4_ESM.tif]

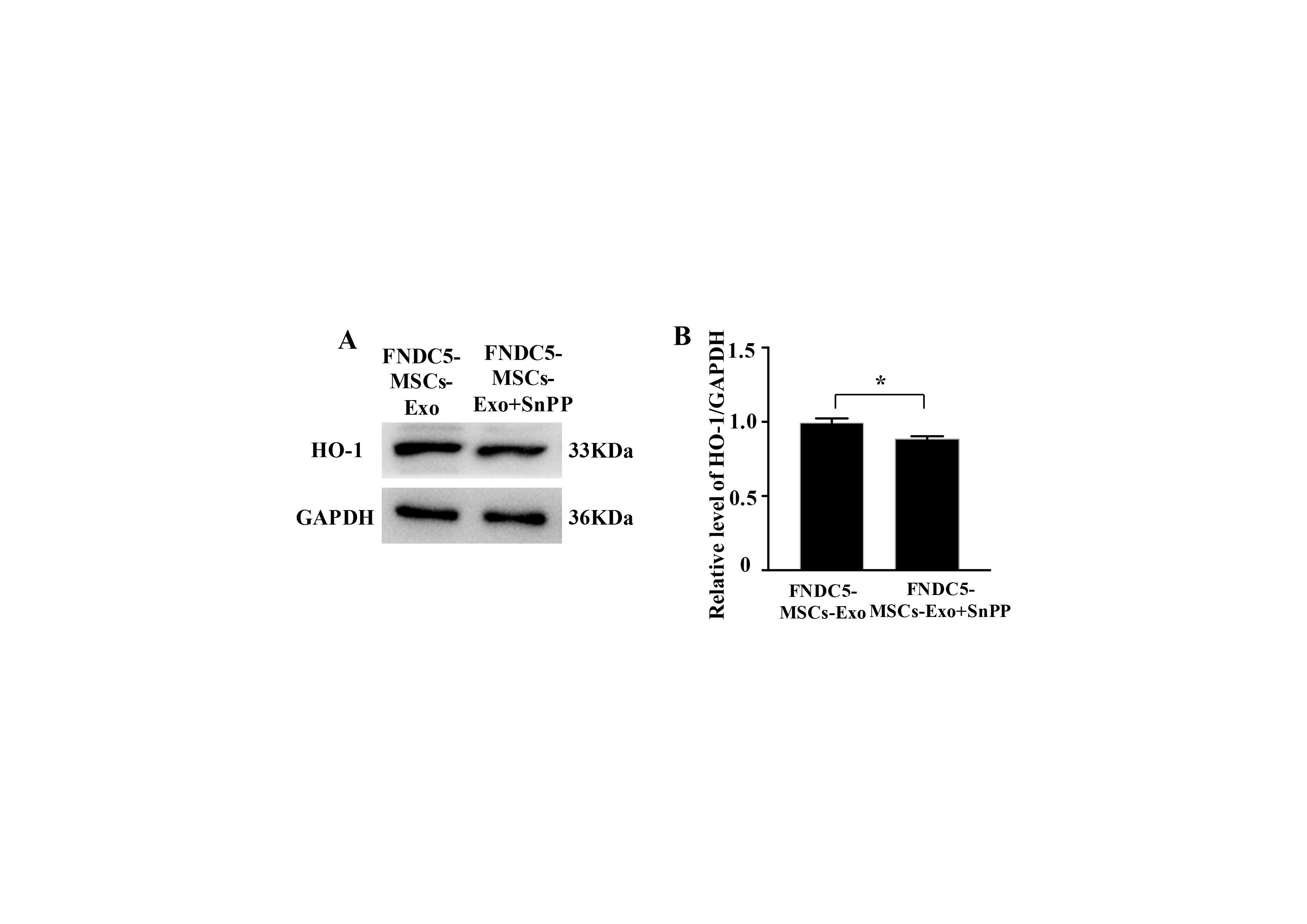

Supplement: Supplementary file 5 — Additional file 5. Figure S5: Expression of HO-1 of Raw264.7 cells with SnPP treatment. A Western blot analysis of HO-1 under different conditions. B Representative semi quantification analysis of HO-1. Data are expressed as the means ± SEM; n = 5; *p < 0.05 [file 13287_2021_2591_MOESM5_ESM.tif]
